# Urbanization Alters Phenology, Mating System Allocation, and Life History of *Impatiens capensis* (Balsaminaceae) via Trait‐Specific Plasticity and Genetic Differentiation

**DOI:** 10.1002/ece3.71583

**Published:** 2025-06-18

**Authors:** Aiden M. Stanley, Tia‐Lynn Ashman

**Affiliations:** ^1^ Department of Biological Sciences University of Pittsburgh Pittsburgh Pennsylvania USA

**Keywords:** common garden, life history, mixed mating system, phenology, plasticity, urbanization

## Abstract

Urbanization is a major human‐mediated driver of environmental change. Plants in urban environments may differ in timing and investment in key life history traits compared to rural plants as a result of genetic differentiation or plastic responses to the urban environment. However, it is unclear for many species whether genetic differentiation or plasticity has shaped urban phenotypes. *Impatiens capensis* is an annual plant that produces self‐pollinating and outcrossing flowers, varying in timing and amount based on environmental conditions. In this study, we characterized differences in floral phenology, mating system allocation, and key life history events between urban and rural populations of *I. capensis* , in situ and in greenhouse common gardens. We asked whether (1) floral investment varies with urbanization in situ, if (2) the differences between urban and rural populations are maintained (genetic differentiation) or lost/exaggerated (plasticity) in a common garden, if (3) differences can be attributed to shifts in life history strategy, and if (4) urban population traits are more variable (higher coefficient of variation). In situ, we found that urban populations advanced flowering time and invested more in outcrossing flowers compared to rural populations. Within greenhouse common gardens, urban plants maintained advanced flowering and were less variable than rural plants (low CV), indicative of genetic differentiation. In contrast, urban plants lost outcrossing bias in mating system allocation observed in situ, indicating plasticity, although both urban and rural plants were highly variable (high CV) for this trait. Early onset of selfing flowers was tied to earlier germination, but outcrossing onset was not affected by germination time. Flowering probability in urban plants was higher than rural ones in common gardens. Our study demonstrates that urbanization influences plant phenotypes through both genetic differentiation and phenotypic plasticity, but the relative importance of the two mechanisms of change vary among floral traits.

## Graphical abstract

Plants in urban environments may differ in timing and investment in key life history traits compared to rural plants, particularly for species with flexible mixed mating system expression. However, it is unclear for many species whether genetic differentiation or plasticity has shaped urban phenotypes. Here, we present evidence supporting trait‐specific influences of genetic differentiation and plasticity on a focal annual species, *Impatiens capensis* , in life history, phenology, and mating system allocation using in situ and greenhouse common garden observations.

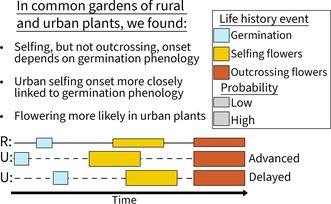

## 1 Introduction

Urbanization is one of the most influential anthropogenic drivers of environmental change (Morris et al. 2020; Orr et al. 2020). Continuous human population expansion necessitates increasing urbanization as the majority of people migrate to cities (Seto et al. 2013). This process requires conversion of terrestrial habitats into impervious surfaces such as asphalt and concrete, which fragments and degrades habitat across the globe (IPCC 2022). One of the most well‐studied changes in urban environments is the Urban Heat Island (UHI) effect which causes urban centers to be 3°C–7°C warmer on average than surrounding rural environments (Tazvali et al. 2015; Liu et al. 2022). Such intense environmental conditions have led to the homogenization of functional diversity, potentially limiting phenotypic expression in urban habitats (Aronson et al. 2014; Groffman et al. 2014; Deguines et al. 2016; Bennett et al. 2020; Silva et al. 2021).

Urbanization across the globe has led to highly replicated selective environmental conditions that may lead to convergent evolution of urban phenotypes, and low variation among such populations (Alberti 2015; Johnson et al. 2015; Thompson et al. 2016; de Barros Ruas et al. 2022; Santangelo et al. 2020, 2022). However, genetic drift, small population size, inbreeding, and reductions in gene flow may instead lead to more diverse phenotypes among urban populations, and high variation among such populations (Johnson and Munshi‐South 2017; Gorton et al. 2018; Miles et al. 2019; Thompson et al. 2022). Moreover, traits may be differentially influenced by urban environmental conditions (reviewed by Johnson and Munshi‐South 2017). That is, urbanization may lead to genetic differentiation for some traits, but plasticity—alteration of trait expression in response to environmental conditions—in others (Jacquemyn et al. 2011; Sotillo et al. 2024). Increased plasticity of trait expression may be favored, as greater responsiveness to environmental fluctuations may lead to higher fitness (Sotillo et al. 2024). However, selection in response to extreme environments can lead to canalized phenotypes that are inflexible to further environmental changes (Lambert et al. 2021).

As sedentary organisms, plants are particularly susceptible to environmental extremes caused by urbanization (Caruso et al. 2019; de Barros Ruas et al. 2022). For example, urbanization has been shown to influence both the timing of life history events (e.g., germination and flowering) and allocation of resources to reproduction (e.g., production of flowers) in many plant species (Ushimaru et al. 2014; Panique and Caruso 2019; Wohlfahrt et al. 2019; Fisogni et al. 2020). In temperate regions, increased temperature due to the UHI effect has been shown to cause earlier emergence in urban habitats compared to conspecifics in rural habitats (Fisogni et al. 2020). Warmer temperatures also tend to advance flowering phenology, either independent of or in conjunction with advances in germination (Sexton et al. 2023), and the latter can lead to even more rapid changes in the schedule of life history events (Wilczek et al. 2010). In contrast to phenology, however, flower production has been shown to decrease in urban habitats that are nutrient poor or pollinator‐limited (Thomann et al. 2013; Rivkin et al. 2020; Rodger et al. 2021). However, some annual plant species invest more in flower production in urban habitats, particularly in response to pollinator rarity, as increased flower production tends to be more attractive to pollinators (Thomann et al. 2013; Panique and Caruso 2019). Moreover, some plant species have the capacity to produce cleistogamous (self‐pollinating, hereafter called “selfing”) flowers that are metabolically cheaper than chasmogamous (hereafter called “outcrossing”) flowers, and allocation to selfing flowers has been shown to increase under unfavorable environmental conditions (Steets et al. 2006; Eckert et al. 2010; Acoca‐Pidolle et al. 2023). Therefore, plants with mixed mating systems that produce both flower types may invest more in selfing flower production in urban habitats, even in spite of an increased risk of inbreeding depression (Eckert et al. 2010; Ushimaru et al. 2014; Rivkin and Johnson 2022; Acoca‐Pidolle et al. 2023). Whether genetic differentiation or plasticity contribute to phenotypic differentiation in urban habitats is an important component of assessing plant resilience to urban stressors (Jacquemyn et al. 2011; Johnson et al. 2015; Lambert et al. 2021).

Coupling in situ observations of urban and rural populations together with observations of their seeds planted in a common garden can tease apart the contribution of genetic differentiation and plasticity to urban plant phenotypes (Lambert et al. 2021; Taichi and Uchimaru 2024). However, it should be cautioned that transgenerational effects may influence phenotypic expression in offspring collected directly from in situ populations (Lambert et al. 2021). Implementing this approach has led to evidence for genetic differentiation, plasticity, or both as drivers of change in response to urbanization (Lambert et al. 2021; Sotillo et al. 2024; Taichi and Uchimaru 2024). In fact, responses to urbanization appear to be trait‐specific. For example, common gardens have revealed genetic differentiation in developmental (e.g., germination) and flowering phenology between urban and rural populations. For instance, urban *Lepidium virginicum* have advanced developmental phenology (Yakub and Tiffin 2017), while urban *Ambrosia artemisiifolia* have advanced flowering phenology (Gorton et al. 2018), but urban *Crepis sancta* have delayed flowering phenology relative to their respective conspecific rural populations (Lambrecht et al. 2016). In contrast, other traits such as plant size and seed production have a plastic response to urbanization, often increasing in urban habitats (Pisman et al. 2020; Ilyas et al. 2021; Sotillo et al. 2024; Taichi and Uchimaru 2024). However, how urbanization influences mating system expression (e.g., ratio of outcrossing to selfing flower production), and whether plasticity plays a role, is still an outstanding question in the field (Suijkerbuijk et al. 2024). Thus, a comprehensive study of multiple traits and phenological milestones is needed to understand how urbanization alters plant phenotypes.

*Impatiens capensis* (Balsaminaceae) is an annual flowering plant that grows in both urban and rural areas of eastern North America and produces both selfing and animal‐pollinated outcrossing flowers (Schemske 1978; Paoletti and Holsinger 2001; Steets et al. 2006; Panique and Caruso 2019). While the flowering phenology of *I. capensis* has shifted earlier in the year due to increasing temperatures in the eastern United States (Bertin et al. 2017; Stanley & Ashman in prep.), it is unknown whether this differs by urbanization extent. Furthermore, although genetic data have indicated that outcrossing flower production is maintained within populations from an urban–rural gradient (Rivkin et al. 2020; Lu 2002; Barker and Sargent 2020; Rivkin and Johnson 2022), outcrossed flowers may not contribute seed to the next generation in unfavorable environments (Steets et al. 2006; Barker and Sargent 2020). In fact, a recent genetic study across multiple cities suggested that selfed seeds may contribute more to the gene pool than outcrossed seeds in rapidly developing urban habitats (Rivkin et al. 2025). Thus, whether floral phenology and mating system allocation by *I. capensis* varies across an urban gradient and whether trait differences between population types reflect plasticity or genetic canalization has yet to be examined.

**FIGURE 1:**
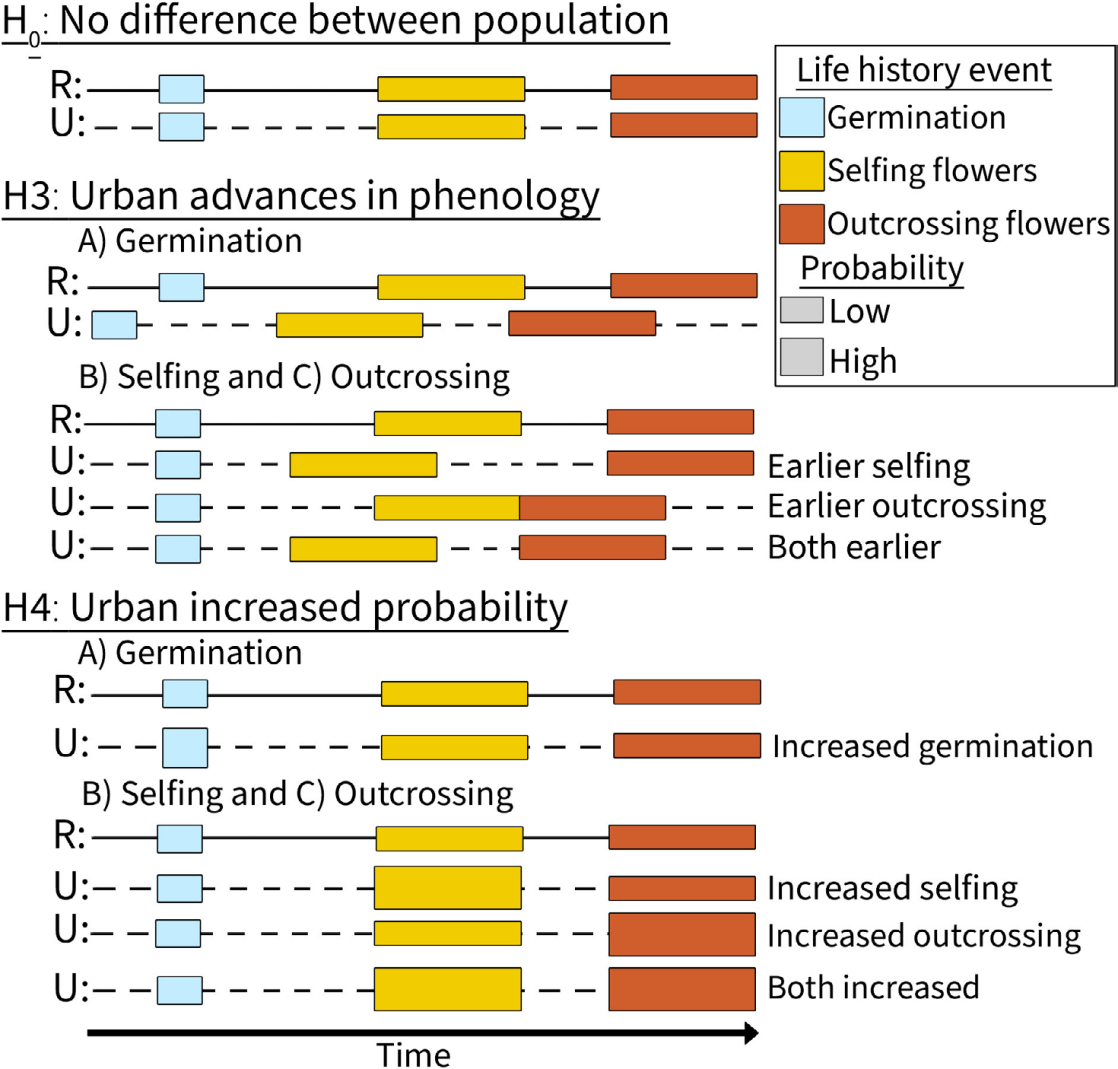
Conceptual diagram of specific hypotheses for **Q3:** How life history events may influence differences found between urban (U, dashed lines) and rural (R, solid lines) phenotypes of *Impatiens capensis* . First, **H**_**0**_ denotes no difference between population types, as the life history cassettes of germination (blue), selfing flower production (yellow), and outcrossing flower production (red) do not differ between urban and rural populations. Next, **H3** hypothesized urban populations have advanced phenology of (A) germination, (B) selfing flower production, and/or (C) outcrossing flower production relative to rural ones. Finally, **H4** hypothesized urban populations have an increased probability (denoted by increased cassette height) of (D) germination, (E) selfing flower production, and/or (F) outcrossing flower production relative to rural ones. Note that hypothesized changes are not mutually exclusive and it is likely that a combination of these strategies are present in urban populations.

In this study, we observed urban and rural populations of *I. capensis* in situ and then in greenhouse common gardens to characterize differences in germination, floral phenology, and mating system allocation. In situ observations captured existing trait variation between urban and rural populations. The use of wild‐collected seeds grown in the standardized environment of greenhouse common gardens allowed us to assess the contribution of canalized factors (genetic and maternal environment) and plasticity to trait variation between rural and urban populations (Lambert et al. 2021; Taichi and Uchimaru 2024). We tested hypotheses for four main questions, specifically, **Q1:** *How do rural and urban populations of* *I. capensis* *differ in flowering phenology and mating system allocation in situ?* We hypothesized that relative to rural populations, urban populations are **H1A:** more phenologically advanced and **H1B:** biased toward greater outcrossing production. **Q2:** *Are observed differences in flowering phenology and mating system allocation maintained in a common garden?* Relative to rural plants, urban plants will **H2A:** maintain advanced phenology and greater outcrossing production, or **H2B:** not express differences in flowering phenology and/or mating system allocation. **Q3:** *Are observed differences in flowering phenology and mating system allocation due to the timing and/or probability of transitioning among key life history events?* Relative to rural ones, urban plants will show **H3:** advanced timing of **(A)** germination, **(B)** selfing, and/or **(C)** outcrossing flowering, and **H4:** greater likelihood of **(A)** germination, **(B)** selfing, and/or **(C)** outcrossing flower production (Figure 1). Finally, we asked **Q4:** *Are urban populations more variable (high coefficient of variation) than rural ones (low coefficient of variation) or vice versa?*

## 2 Materials and Methods

### 2.1 Study System

Orange Jewelweed ( *Impatiens capensis* , Balsaminaceae) is an annual late‐summer flowering plant native to eastern North America. It grows in moist soils across urban and rural habitats, including floodplain forests, riparian edges, and drainage ditches (Antlfinger 1986; Zhao and Schoen 2022). Seeds overwinter and begin to germinate in early Spring. However, they do not persist in the seed bank beyond the next year, meaning that annual seed production is directly responsible for the next generation (Schemske 1978). Importantly, the ratio of selfing and outcrossing flowers produced by an individual is highly environment‐dependent. That is, a favorable environment promotes greater allocation to outcrossing, whereas stressful environments promote selfing flower production (Schemske 1978; Paoletti and Holsinger 2001; Steets et al. 2007; Zhao and Schoen 2022). Outcrossing flowers produce, on average, three to seven seeds per pod on long pedicels, and selfing flowers produce, on average, one to three seeds per pod on short pedicels close to the main stem (Steets et al. 2007). Once matured, seed pods dehisce explosively but leave behind distinct pedicels allowing characterization as selfed or outcrossed seed pods (Schemske 1978).

### 2.2 Study Sites

**FIGURE 2:**
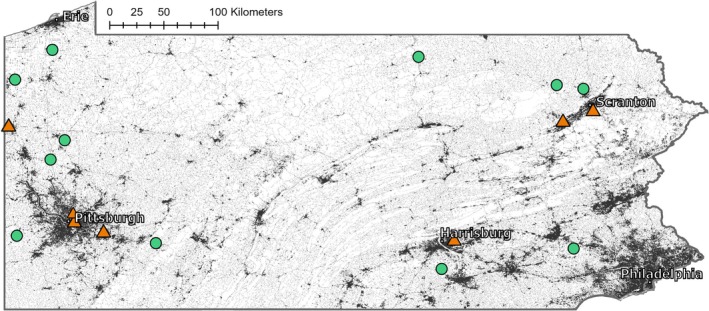
Populations of *I. capensis* characterized in Pennsylvania USA. Populations were categorized as rural (green circles) or urban (orange triangles). Impervious surface is shown as dark gray pixels and all other land use types are shown as white pixels on the map. Thus, city centers like Pittsburgh are represented by dense clusters of dark gray pixels (ESRI 2024).

Eighteen wild populations of *I. capensis* were characterized across the state of Pennsylvania, USA (Figure 2; Appendix A, Table A1). The 2019 National Landcover Database (Wickham et al. 2023) was used to quantify the amount of impervious surface in a 5 km radius around each population centroid with a 30 × 30 m resolution in ArcGIS Pro (v2.6.3; ESRI 2024), as impervious surface has been shown to be an important indicator of urbanization for plant ecology (Yan et al. 2019). Each population was categorized as either rural (< 15% impervious surface) or urban (> 45% impervious surface; as reviewed by Wenzel et al. 2020). This was repeated for 2.5 km, 500 m, and 100 m radii around each population, with the same outcome for categorizing each site. When possible, populations were chosen on the basis of minimizing other habitat differences (e.g., similar canopy cover and water availability). All sites were at least 4 km apart, but a minimum linear distance of 10 km apart was used to ensure rural and urban populations did not share primary pollinators, thus limiting the likelihood that intermixing occurred between population types (Emel et al. 2020; Rivkin and Johnson 2022; Mola and Williams 2025).

### 2.3 In Situ Observations

Each population was surveyed twice during the flowering season of August–October 2021. Surveys conducted between August 20 and September 12 were considered “early”, and surveys that occurred between September 24 and October 17 were considered “late”. At each population, 10 plants were chosen at random along a 20 m transect through the center of the population. For each plant, the number of selfing and outcrossing flowers, seed pods, and pedicels were recorded. Flowering phenology was scored on a phenophase scale of 0–8 (e.g., Pearson 2019; Lu et al. 2023), where lower numbers represent earlier stages such as vegetative only (Stage 0) first selfing flower (Stage 1) and first outcrossing flower buds (Stage 2), and later stages represent peak flowering (Stage 5), fruit production (Stage 6), and total senescence (Stage 8). Mating system allocation was calculated as the ratio of total outcrossing to total selfing structures (i.e., buds, flowers, seed pods, and pedicels) on a given plant. A value greater than one represents a greater production of outcrossing over selfing flowers, and less than one represents higher selfing production. To account for differences in plant size at sampling, plant height was measured from the first node above soil level to the tip of the highest branch, rather than to the apical meristem which is often lost to browsing herbivores (Steets et al. 2006).

### 2.4 Common Garden Observations

#### 2.4.1 Germination Timing and Probability

For greenhouse common gardens, we used selfed seeds collected across 10 in situ populations in early October of 2022 and 2023. In 2022, seeds were collected from up to 20 randomly selected mothers per population (average 113, range 40–201; Appendix A). In 2023, seeds were collected from 12 randomly selected mothers per population (average 121, range 119–123; Appendix A). Seeds from different mothers were pooled to evaluate population‐level differences. A total of 1130 and 1208 seeds were collected during 2022 and 2023, respectively. Each seed was sterilized in 5% bleach before being placed individually in the well of a 48 well‐plate and submerged in 300 mL of distilled water, which was changed weekly to control for fungal growth. Seeds were cold‐stratified at 4°C in this manner for approximately 4 months (per Steets et al. 2006). The day of radicle emergence from the seed was recorded as germination date. Seeds were observed initially 1× weekly, then 3× weekly after the first seed germinated in early January. Germination was considered complete when no more seeds had germinated for a consecutive three‐week period, in mid‐March of both years. Germination probability was scored as a binary metric, 0 if the seed did not germinate, or 1 if it did.

#### 2.4.2 Flowering Onset and Probability

In each year, a randomly chosen subset of germinated seeds per population was planted individually in Jolly Gardener Germination Mix soil (BFG Supply) in 72‐cell propagation trays and allowed to grow for approximately 3 weeks in growth chambers under a 12:12 h light cycle, 12°C–16°C, 30%–50% relative humidity. Afterward, they were transplanted to 6″ pots in Jolly Gardener C/20 soil (BFG Supply), fertilized using Plantone 2:1:1, and moved into a common garden in the greenhouse at the University of Pittsburgh in Pennsylvania, USA. Greenhouse conditions were a 16:8 h light cycle, 15°C–28°C, and 30%–50% RH conditions. Plant location in both the growth chamber and greenhouse spaces was completely randomized. In the 2022 cohort, 20 plants from each of 10 populations were randomly selected, for a total of 200 plants. In the 2023 cohort, 18–26 plants from each of 10 populations were randomly selected, for a total of 213 plants. Plants were kept moist and fertilized as needed throughout the study. Each plant was examined twice weekly for the presence of selfing and outcrossing structures, and the production of the first selfing and first outcrossing flower was recorded as the onset of the respective flowering type. The number of days from germination to flower production was calculated for each flower type on each plant. Production of selfing and outcrossing flowers was scored separately for each plant as a binary metric: 0 denoting a (selfing or outcrossing) flower was not produced, or 1 if it was. In addition to these metrics, we also calculated flowering phenology and outcrossing to selfing ratio for early and late surveys, similar to in situ observations. We pooled data from the first half of the greenhouse experiment to comprise the early survey (April–May), and data from the second half to comprise the late survey (June–July).

### 2.5 Analyses

All analyses were performed in R Statistical Software (v.4.3.3; R Core Team 2025) and used generalized linear mixed models (GLMMs) from the lme4 (v1.1–31) package. Pseudo R‐squared values for model fit of GLMMs were calculated with the *r.squaredGLMM* function from the MuMIn (v1.47.5) package. Model selection via the *drop1* function was used to compare the models with and without each fixed effect, based on the Akaike information criterion (AIC; Aho et al. 2014), and we chose the best fitting ecologically relevant model based on a cutoff of < Δ2 from the lowest AIC scoring model. Each model contained population type (urban or rural) as a fixed effect, and population ID as a random effect to account for within‐population type differences. To account for differences between maternal environments across the 2 years that seeds were collected from wild populations, phenology and probability of germination, selfing, and outcrossing in common gardens included a fixed effect of seed collection year and the interaction of population type*seed collection year. Germination phenology and probability from collection included an additional random effect of well‐plate ID, to account for possible variation due to differences in microclimate during cold stratification. Plants that did not germinate, or produce selfing or outcrossing flowers, were removed from the respective phenology analyses. Probability of germination, selfing, and outcrossing flower production were assessed in separate GLMMs with a binomial distribution. The models for flowering phenology and mating system allocation both in situ and in greenhouse common garden included the additional factors of survey (early or late), survey*population type interaction, and the covariate plant height. Post hoc planned contrasts with Bonferroni corrections for multiple comparisons were performed for significant interactions of population type*survey and population type*year, to evaluate differences between population types within‐survey and within‐year, as well as within‐population types between surveys and years (Appendix B) using the *emmeans* function from the emmeans (v1.8.9) package. To investigate differences in variation between urban and rural populations, we used the *cv* function from the raster (v3.6–26) package to produce the coefficient of variation from the population means within population type between surveys for flowering phenology and mating system allocation, and a Modified signed‐likelihood ratio test using the *mslr_test2* function from the cvequality package (v0.2.0; Marwick and Krishnamoorthy 2019) was used to determine significant differences between the groups (Pélabon et al. 2020).

## 3 Results

### 3.1 How Do Rural and Urban Populations of *I. capensis* Differ in Flowering Phenology and Mating System Allocation In Situ?

**FIGURE 3:**
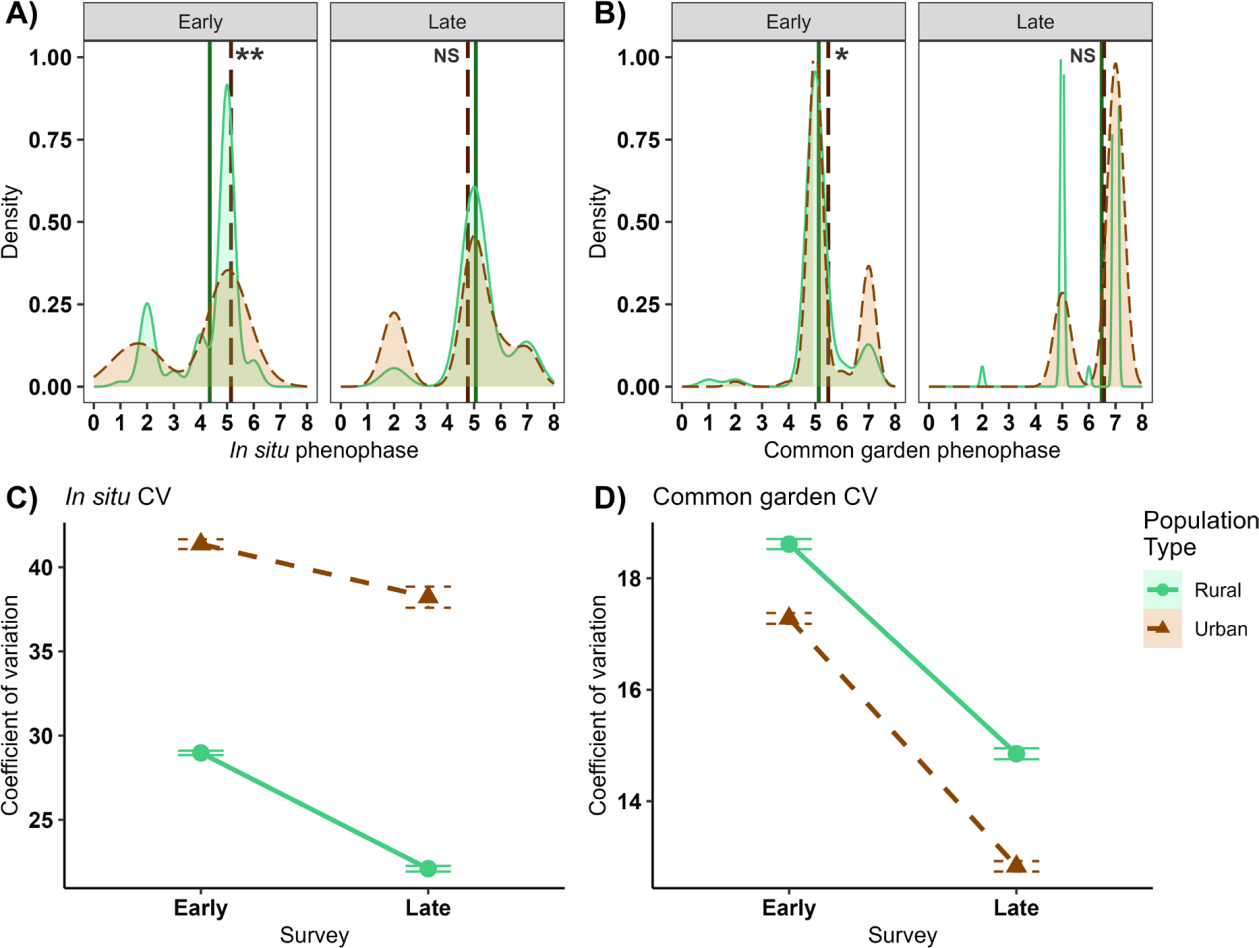
Density plots with means (vertical lines) of flowering phenology of rural (green solid) and urban (orange dashed) populations (A) in situ and in (B) greenhouse common garden, surveyed early (left panel) and late (right panel) in the flowering season. In both studies, urban populations were significantly more advanced than rural ones in the early, but not late survey. Rural populations were less variable in flowering phenology than urban ones, but both shifted toward senescence in the late survey. (C) Coefficient of variation (CV) ± SE for in situ flowering phenophase was higher in urban populations than rural ones; CV decreased in both populations in the late survey, but more sharply in rural populations. (D) CV ± SE for greenhouse common garden plants was higher for rural plants than urban ones, indicating greater environmental sensitivity in urban plants, although CV declined for both population types in the late survey. Note the different y‐axis ranges for in situ and common garden CVs. Flowering phenology was measured as a phenophase scale of one to eight that ranged from non‐flowering vegetative (i.e., 0), peak flower production (i.e., 5), to fruiting and senescence (i.e., 8). NS *p* > 0.05, *p < 0.05, **p < 0.01.

Flowering phenology of *I. capensis* differed significantly by the population type*survey interaction in situ (*p* = 0.04; Table 1A). Post hoc analysis of flowering phenology revealed that in the early season survey, urban populations were more advanced, having already reached peak flowering, when compared to rural populations that had more outcrossing buds than open flowers (mean phenophase: 4.95 ± 0.25 vs. 4.35 ± 0.12, respectively; Appendix B, Table A2). In the late season survey, however, urban and rural populations overlapped in phenology, with rural populations having reached peak flowering (mean phenophase: 5.07 ± 0.19) and urban populations maintaining peak flowering (mean phenophase: 4.76 ± 0.41; Figure 3A). There was a significant effect of population type on flowering phenophase (*p* = 0.007), in which flowering phenology was more advanced in urban populations than rural ones. Individuals of both populations senesced from the early (4.74 ± 0.15) to the late survey (4.91 ± 0.20, *p* = 0.0007). Across all plants, increased height advanced flowering phenology (i.e., larger plants flowered earlier in the season; *p* = 0.009). In situ flowering phenology was more variable in early surveys of urban populations (mean CV: 41% ± 0.29%) than rural ones (mean CV: 29% ± 0.1%), but variation decreased in late surveys more sharply in rural (mean CV: 22% ± 0.2%) than urban populations (mean CV: 38% ± 0.6%, *p* = 0.007; Figure 3C).

**TABLE 1:** Fixed effects for LMMs of urban and rural (A) in situ populations and (B) greenhouse *I. capensis* phenophase in surveys of the early and late flowering season.

Study	Parameters	*X* ^2^	df	*p*
(A) In situ	Population type	7.282	1	0.007
Pseudo‐*R* ^2^ = 0.136	Survey	11.41	1	0.0007
*N* = 168	Population type*survey	4.232	1	0.04
	Plant height	6.884	1	0.009
(B) Common garden	Population type	7.737	1	0.005
Pseudo‐*R* ^2^ = 0.315	Survey	97.25	1	< 0.0001
*N* = 388	Population type*survey	1.995	1	0.2
	Plant height	0.097	1	0.8
	Germination day	1.206	1	0.2

*Note:* Phenophase was measured on a scale of 0–8 (0 = vegetative, 1 = first selfing flower, 2 = first outcrossing flower, 5 = peak flowering, 6 = fruit production, 8 = senescence). Pseudo‐*R* ^2^ and sample size (*N*) are shown beneath the response variable for each analysis.

**FIGURE 4:**
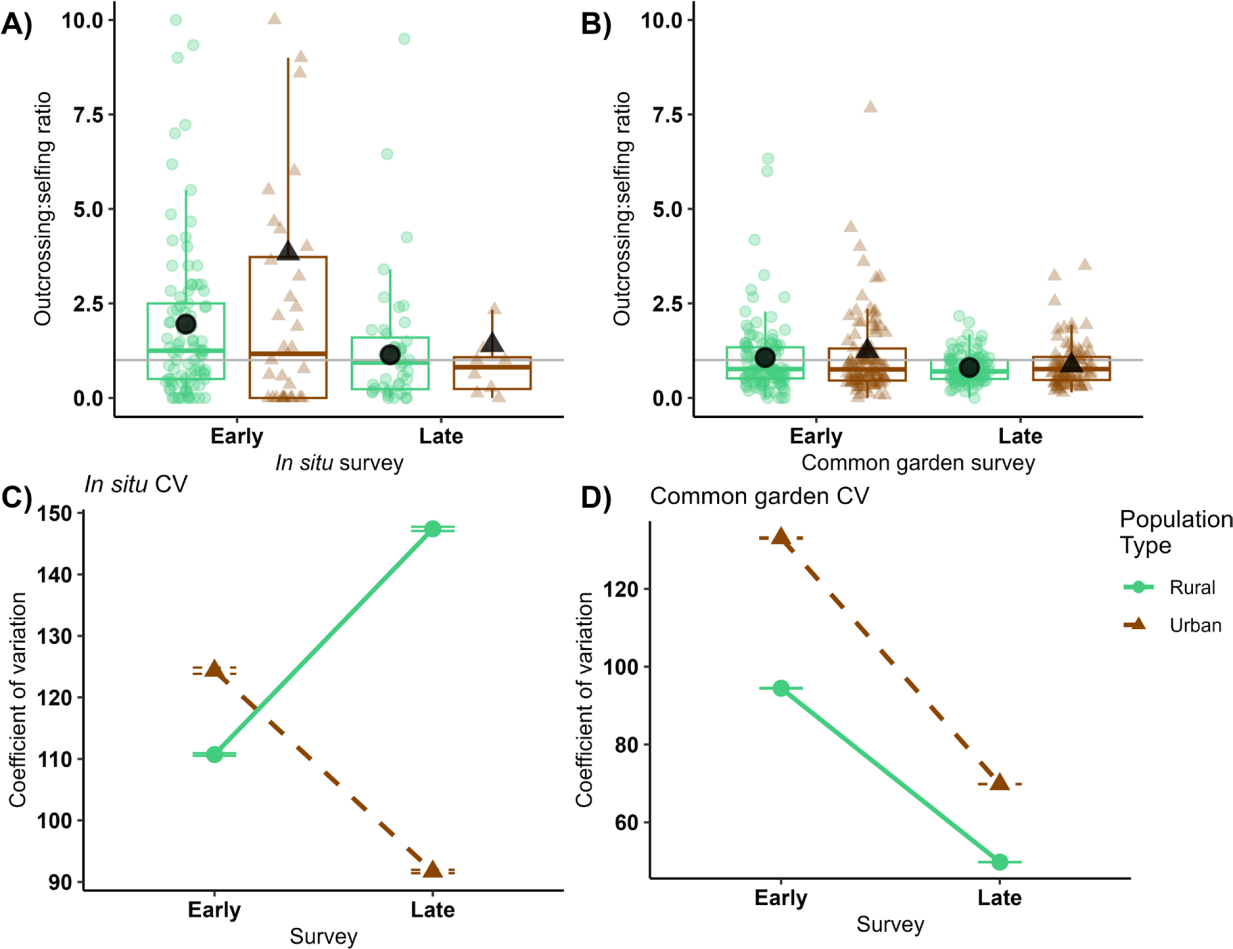
Outcrossing to selfing ratio displayed as boxplots overlayed with raw data for rural (green circles) and urban (orange triangles) populations in (A) in situ and (B) greenhouse common garden of plants surveyed early (left panel) and late (right panel) in the flowering season. Estimated marginal means are shown as large black symbols. (C) CV ± SE of outcrossing to selfing was not significantly different for in situ urban and rural populations, rather it was highly variable overall. (D) CV ± SE of outcrossing to selfing ratio was higher in urban than in rural plants in the greenhouse common garden, though CV declined for both plant types between the early and late surveys. Note the different y‐axis ranges for CVs.

In situ mating system allocation was more biased toward greater outcrossing production in urban populations when compared to rural ones (outcrossing to selfing flowers 2.6 ± 0.6 vs. 1.5 ± 0.4, respectively, *p* = 0.001; Table 2A). Outcrossing to selfing ratio decreased from the early to late survey in both populations (*p* = 0.03, Figure 4A), without a significant interaction of population type*survey (*p* = 0.1). Plant height was positively correlated with outcrossing to selfing ratio (i.e., larger plants produced more outcrossing than selfing flowers, *p* < 0.0001). The coefficient of variation for outcrossing to selfing ratio was not significantly different between population types (*p* = 0.76), surveys (*p* = 0.31), or by the population type*survey interaction (*p* = 0.56), but was high overall (mean CV: 123 ± 0.17, Figure 4C).

**TABLE 2:** Fixed effects for linear mixed models of outcrossing to selfing ratio of urban and rural (A) in situ populations and (B) greenhouse *I. capensis* in surveys during the early and late flowering season.

Study	Parameters	*X* ^2^	df	*p*
(A) In situ	Population type	10.33	1	0.001
Pseudo‐*R* ^2^ = 0.319	Survey	4.935	1	0.03
*N* = 168	Population type*survey	2.285	1	0.1
	Plant height	37.39	1	< 0.0001
(B) Common garden	Population type	0.635	1	0.4
Pseudo‐*R* ^2^ = 0.182	Survey	3.074	1	0.08
*N* = 391	Population type*survey	0.332	1	0.6
	Plant height	0.026	1	0.9

*Note:* Pseudo‐*R* ^2^ and sample size (*N*) are shown beneath the response variable for each analysis.

### 3.2 Are Observed Differences in Flowering Phenology and Mating System Maintained in a Common Garden?

When grown in the greenhouse common garden, flowering phenology was more advanced in urban plants than rural ones; on average, urban plants matured fruits when rural ones were still transitioning from peak flowering to fruiting (phenophase 6.03 ± 0.08 vs. 5.80 ± 0.08, respectively, *p* = 0.005; Table 1B). Plants from both populations senesced from the early to the late survey (*p* < 0.0001, Figure 3B) at the same pace (i.e., no population type*survey interaction, *p* = 0.27). Across all populations, neither time to germination nor plant height significantly influenced flowering phenology (*p* = 0.2 and *p* = 0.8, respectively). CV of phenophase was significantly lower in urban plants in both the early (17.3 ± 0.1) and late survey (12.8 ± 0.09) when compared to rural plants in the early (18.6 ± 0.09) and late survey (14.9 ± 0.1, *p* = 0.003; Figure 3D).

On average, mating system allocation in the greenhouse common garden did not differ between urban and rural populations (*p* = 0.4; Table 2B). Mating system allocation differed only marginally between early and late surveys of the common garden plants, due to the overall low outcrossing to selfing ratio (mean 0.99 ± 0.05, *p* = 0.08, Figure 4B), without a significant interaction between population type and survey (*p* = 0.6). Plant height did not significantly influence mating system allocation (*p* = 0.9). CV of outcrossing to selfing ratio was significantly higher in urban plants in both the early (133 ± 0.16) and late survey (69.8 ± 6) when compared to rural plants in the early (94.5 ± 0.09) and late surveys (49.8 ± 0.04, *p* < 0.0001; Figure 4D).

### 3.3 Are Observed Differences in Flowering Phenology and Mating System Allocation due to the Timing and/or Probability of Transitioning Among Key Life History Events?

#### 3.3.1 Germination Probability

**FIGURE A1:**
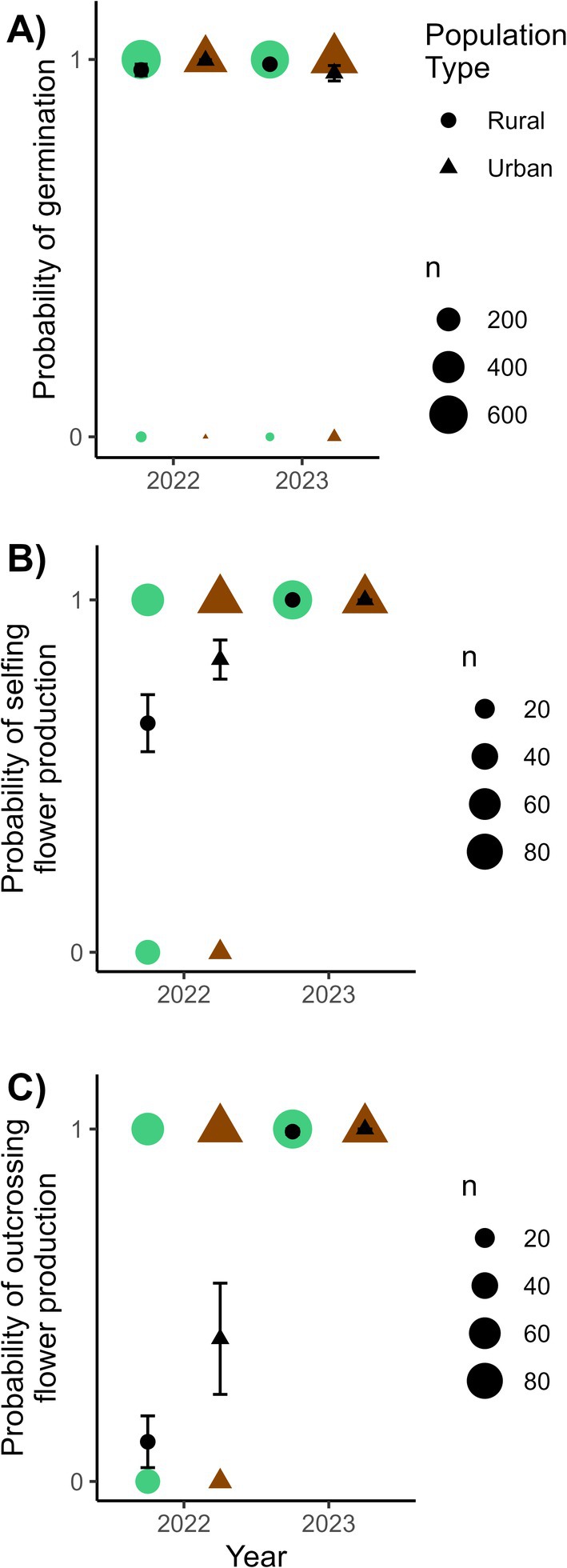
Binomial data of probability of (A) germination, (B) selfing flower production, and (C) outcrossing flower production for rural (green circles) and urban (orange triangles) populations of *I. capensis* in common gardens, where size of points is scaled by number of plants with that value. Estimated marginal means (±SE) are shown as black symbols with error bars. The probability of germination was very high, but differed slightly between population types, years, and their interaction (Table 3A, main text). Probability of selfing was higher in urban plants (*p* = 0.05), but did not differ between years or by the population type*year interaction (Table 3B, main text). Outcrossing flower production differed significantly between years (*p* < 0.0001), but did not vary significantly between population types (*p* = 0.09) or the population type*year interaction (Table 3C, main text).

Overall, there was a high probability of germination in both years (range 96%–100%). Germination probability was significantly affected by the population type*year interaction (*p* < 0.0001, Table 3A). Post hoc analysis revealed that seeds from urban populations germinated 100% in 2022 and 96% ± 2% in 2023, whereas rural seeds germinated 98% ± 1% across both years (Appendix B, Table A3; Appendix C, Figure A1A). Across years, urban seeds were 1% more likely to germinate than rural ones (*p* = 0.01), and across population types, seeds collected in 2022 were 1% more likely to germinate than those in 2023 (*p* = 0.02).

**TABLE 3:** Results from binomial GLMMs for probability of (A) germination, (B) selfing flower production, and (C) outcrossing flower production of *I. capensis* seeds originating from in situ urban and rural populations in Pennsylvania USA, and grown in greenhouse common gardens.

Response variable	Parameters	*X* ^2^	df	*p*
(A) Germination	Population type	6.393	1	0.01
Pseudo‐*R* ^2^ = 0.418	Year	5.108	1	0.02
*N* = 2338	Population type*year	26.32	1	< 0.0001
(B) Selfing flower	Population type	3.786	1	0.05
Pseudo‐*R* ^2^ = 0.968	Year	0.002	1	0.9
*N* = 375	Population type*year	0.000	1	0.9
(C) Outcrossing flower	Population type	2.909	1	0.09
Pseudo‐*R* ^2^ = 0.983	Year	44.52	1	< 0.0001
*N* = 375	Population type*year	0.000	1	0.9

*Note:* Seeds were collected in two different years, 2022 and 2023. Pseudo‐*R* ^2^ and sample size (*N*) are shown beneath the response variable for each analysis.

#### 3.3.2 Germination Phenology

**FIGURE 5:**
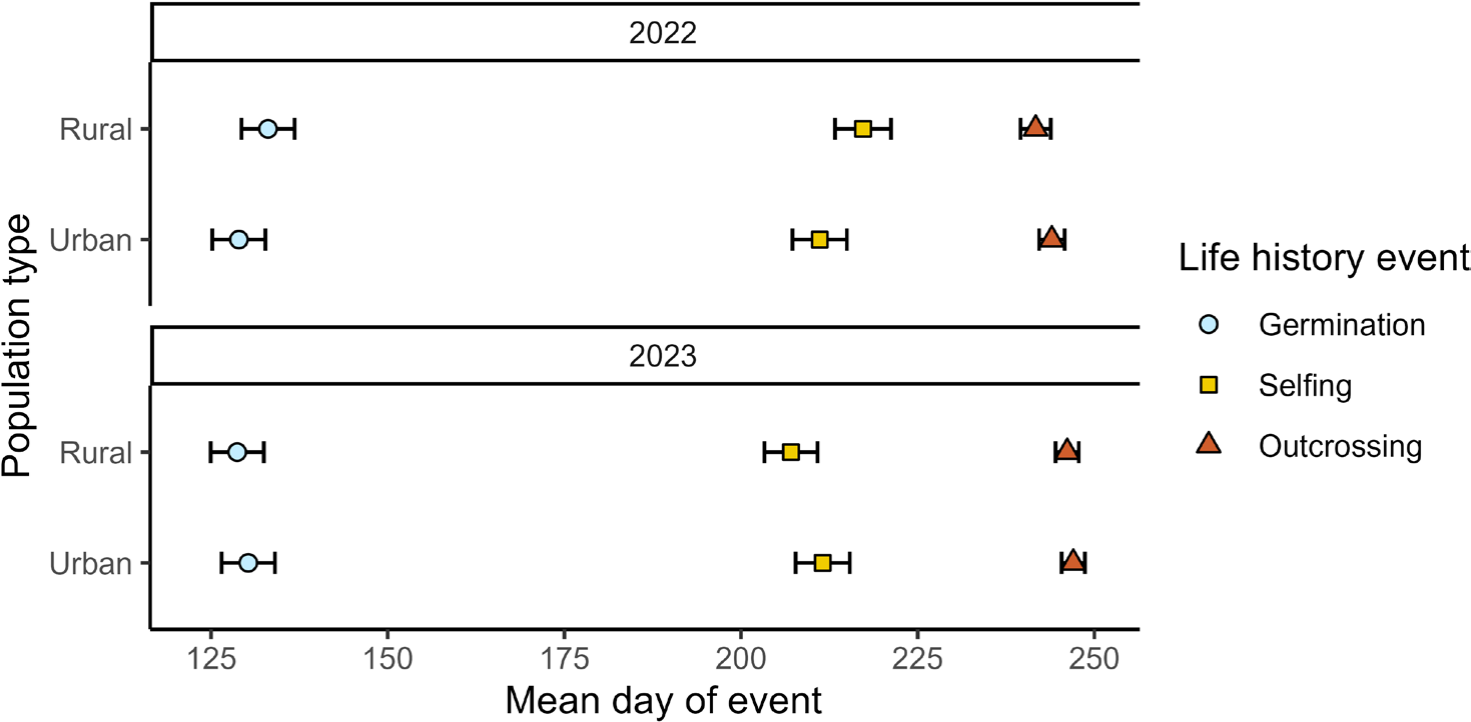
Estimated marginal means (±SE) onset of key life history events for urban and rural plants grown in greenhouse common gardens in 2022 and 2023. When germination (circles) occurred earlier in the year, the timing of selfing onset (squares) was also more advanced, occurring earlier in the year. When germination was more delayed, the selfing onset also shifted later in the year. However, the timing of outcrossing onset (triangles) did not shift significantly with any directional changes in germination or selfing flower production. Interestingly, urban plants displayed a heightened sensitivity to developmental phenology shifts in comparison to rural ones (i.e., earlier germination led to even earlier selfing onset in urban populations).

The average number of days to germination from seed collection varied between years (population type*year interaction *p* < 0.001; Table 4A). Post hoc analysis showed that in 2022, seeds collected from urban populations germinated 3 days earlier than rural ones (127 ± 3 vs. 130 ± 2 days post‐collection, respectively; Figure 5; Appendix B, Table A4). In 2023, however, seeds collected from urban populations germinated at the same time as rural ones (132 ± 3 vs. 130 ± 3, respectively). While there was also a significant effect of year, in which 2023 seeds were delayed by 2 days compared to 2022 (*p* = 0.03), there was not a significant effect of population type alone (*p* = 0.2).

**TABLE 4:** Results from LMMs for timing of (A) germination, (B) selfing flower onset, and (C) outcrossing flower onset of *I. capensis* originating from seeds collected from in situ urban and rural populations in Pennsylvania, USA and grown in greenhouse common gardens.

Response variable	Parameters	*X* ^2^	df	*p*
(A) Germination	Population type	1.512	1	0.2
Pseudo‐*R* ^2^ = 0.380	Year	4.729	1	0.03
*N* = 2258	Population type*year	84.08	1	< 0.0001
(B) Selfing flower	Population type	0.489	1	0.5
Pseudo‐*R* ^2^ = 0.376	Year	0.183	1	0.7
*N* = 322	Population type*year	4.397	1	0.04
(C) Outcrossing flower	Population type	0.120	1	0.7
Pseudo‐*R* ^2^ = 0.690	Year	0.046	1	0.8
*N* = 237	Population type*year	0.104	1	0.7

*Note:* Seeds were collected in two different years, 2022 and 2023. Pseudo‐*R* ^2^ and sample size (*N*) are shown beneath the response variable for each analysis.

#### 3.3.3 Flowering Probability

When grown in common gardens in the greenhouse, 90% ± 6% of urban plants produced selfing flowers in comparison to 82% ± 8% of rural ones (*p* = 0.05). In contrast, the probability of a plant producing a selfing flower in its lifetime did not differ between years or by the population type*year interaction (Table 3B, Appendix C, Figure A1B). With respect to outcrossing flowers, urban plants were more variable in the probability of producing them than rural ones (68% ± 16% vs. 59% ± 7%), thus the mean difference was not significant (*p* = 0.09, Table 3C, Appendix C, Figure A1C). The probability of producing an outcrossing flower was 100% in 2023, but only 23% ± 9% in 2022 (*p* < 0.0001). However, outcrossing flower production did not differ between population types within different years (population type*year interaction, *p* = 0.99).

#### 3.3.4 Flowering Onset

In common gardens, urban and rural plants differed in the time to selfing onset (i.e., number of days between germination and first selfing flower), but this varied by year (population type*year *p* = 0.04; Table 4B). Post hoc analysis showed that, while not significant, in 2022 urban plants produced selfing flowers earlier than rural plants (78 ± 7 vs. 84 ± 7 days, respectively; Figure 5) and in 2023, urban plants produced selfing flowers slightly later than rural plants (84 ± 7 days vs. 81 ± 7 days, respectively; Appendix B, Table A5). There was neither a significant main effect of population type (*p* = 0.5) nor year (*p* = 0.7) in the number of days between germination and first selfing flower. The mean time to first outcrossing flower from germination was 117 ± 1 days, and there was no effect of population type (*p* = 0.73), year of seed collection (*p* = 0.83), or their interaction (*p* = 0.75, Table 4C; Figure 5).

## 4 Discussion

**FIGURE 6:**
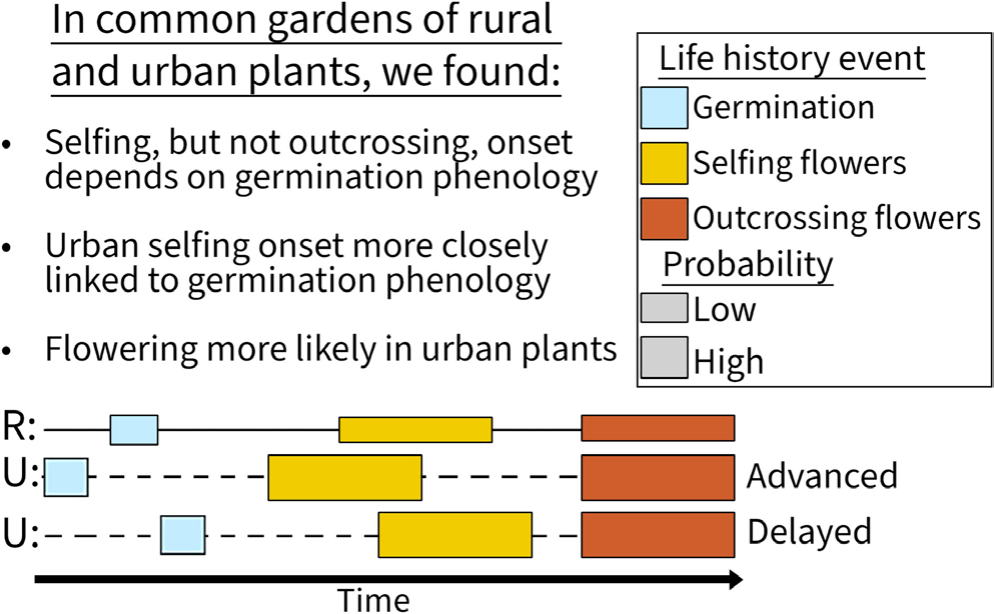
Summary of results following hypothetical Figure 1 in which common gardened urban and rural plants showed differences in germination, selfing, and outcrossing probability, and shift timing of selfing onset as a result of shifting germination timing. This shift in selfing phenology is more pronounced in urban than in rural plants. In contrast, outcrossing is unresponsive to timing of germination or selfing, and instead responds to contemporaneous environmental cues.

Urbanization can lead to strong selection on plant phenotypes, leading to convergent evolutionary trajectories in urban populations that are distinct from rural ones (Alberti 2015; Johnson et al. 2015; Thompson et al. 2016; de Barros Ruas et al. 2022; Santangelo et al. 2020, 2022). Our use of multiple populations from across a range of latitude and longitude in Pennsylvania, USA (Figure 2) allowed us to generalize the effects of urbanization on plant phenotypes more broadly beyond the influence of local adaptation (e.g., Thompson et al. 2016; Lambert et al. 2021; Taichi and Uchimaru 2024; Rivkin et al. 2025). Our results demonstrated that urbanization alters the phenology, mating system allocation, and life history of *I. capensis* in trait‐specific and growth environment‐dependent capacities. The effect of urbanization on mating system allocation varied between in situ and greenhouse common garden, suggesting a strong role for plasticity, while differences between urban and rural populations in the probabilities of germination and flower production, and aspects of flowering phenology were retained in the greenhouse, indicative of genetic differentiation. Additionally, we found that the onset of selfing flowering covaried strongly with advanced germination in both population types, but this was not the case for the onset of outcrossing flowering (Figure 6). Although we note that the differentiation between population types could include transgenerational effects in addition to genetic ones, because we did not produce and compare offspring phenotypes within the greenhouse (Lambert et al. 2021). Nevertheless, the parallel phenotypic changes in all five of our urban populations relative to our five rural ones suggest strong differentiation in phenotypic expression due to urbanization (e.g., Taichi and Uchimaru 2024). Below we discuss how these changes can inform on phenotypic patterns found in urban and rural populations and the implications for plasticity versus genetic differentiation as drivers of urban and rural differences.

### 4.1 How Do Rural and Urban Populations of *I. capensis* Differ in Flowering Phenology and Mating System Allocation In Situ?

There is a large body of evidence supporting earlier flowering in situ in urban populations relative to their rural counterparts, particularly in temperate regions of the Northern Hemisphere (Neil and Wu 2006; Yakub and Tiffin 2017; Gorton et al. 2018; Wohlfahrt et al. 2019; Sexton et al. 2023). In contrast, fewer studies have examined the influence of urbanization on mating system allocation, and how mating system expression in mixed‐mating species is affected is still an outstanding question in urban research (Suijkerbuijk et al. 2024). Research on a few species' mating system allocation in urban settings has found varied responses. There is, however, a growing consensus that pollination services tend to be lacking in urban environments, leading to reduced seed set in many outcrossing species (e.g., Eckert et al. 2010; Harrison and Winfree 2015; Bennett et al. 2020; de Barros Ruas et al. 2022). For example, the mixed‐mating *Portulaca oleracea* was found to predominantly produce only selfing individuals in urban populations, whereas rural populations were a mix of selfing and outcrossing individuals (Fujita et al. 2025). Other species in urban settings, such as self‐compatible annuals *Crepis sancta* and *Commelina communis* , were found to have increased selfing rates primarily due to reduced pollinator availability (Cheptou and Avendaño 2006; Ushimaru et al. 2014). Self‐compatible species, such as the perennial *Gentiana dahurica*, increase outcrossing flower production in urban systems (Hou et al. 2019), but self‐incompatible species, such as the perennial *Turnera subulata*, have been shown to be more strongly influenced by pollen limitation than overarching urban effects (Serafim et al. 2025). Interestingly, our focal species in its Canadian range was found to maintain similar outcrossing rates between urban and rural populations (Lu 2002; Barker and Sargent 2020; Rivkin and Johnson 2022). In our study, we found similarity in phenotypes across a range of urban populations in Pennsylvania, USA and these included advanced peak flowering phenology (Figure 3A) and a greater ratio of outcrossing to selfing flowers when compared to rural populations (Figure 4A). While flowering phenology was only somewhat variable across populations (Figure 3C), mating system allocation was highly variable (Figure 4C), which may be due to contemporaneous abiotic (e.g., stress; Suijkerbuijk et al. 2024) or biotic (e.g., conspecific density; Schmitt et al. 1987) differences among populations. Adding our results to the existing body of evidence suggests that there is no universal evolutionary trajectory of mating system in response to urbanization but rather that multiple evolutionary pathways are possible, perhaps reflecting more nuanced differences in urban settings.

### 4.2 Are Observed Differences in Flowering Phenology and Mating System Allocation Maintained in a Common Garden?

Although advanced flowering phenology in urban populations is common (Neil and Wu 2006; Yakub and Tiffin 2017; Gorton et al. 2018; Wohlfahrt et al. 2019; Sexton et al. 2023), flowering phenology is also known to be very sensitive to environmental cues and respond plasticly to increased temperature due to the UHI effect (Neil and Wu 2006). Few prior studies determined that differences between urban and rural flowering phenology are a result of genetic differentiation (however, see Lambrecht et al. 2016; Géron et al. 2024; Woudstra et al. 2024). Removal of variable environmental conditions experienced in situ by growth under uniform greenhouse environments allowed us to uncover evidence supporting both genetic differentiation and plasticity in flowering phenology. In our common garden, urban plants recapitulated the advanced peak flowering phenology observed in our in situ urban populations compared to rural ones. In contrast to in situ observations, however, urban plants in the common garden were less variable in flowering phenology than rural ones (Figure 3B), indicating that flowering phenology may additionally be responding plasticly to contemporaneous cues (similar to Williams et al. 2008).

Although there is a vast body of literature on mating system expression, attention has only recently been given to the influence of environmental change on mating system plasticity (reviewed in Suijkerbuijk et al. 2024). In its Canadian range, Barker and Sargent (2020) found *I. capensis* mating system plasticity in both urban and rural populations, where hand‐pollination uniformly increased investment in outcrossing over selfing flowers. In contrast with in situ observations, but in line with Barker and Sargent (2020), mating system allocation in our greenhouse plants was more biased toward selfing than outcrossing, regardless of population origin (Figure 4B). However, we excluded insect pollinators and did not supplement our plants with hand pollination, which, along with our greenhouse conditions, likely favored a lower investment in outcrossing (Panique and Caruso 2019). This suggests that mating system allocation is more responsive to contemporary conditions rather than long‐term shifts in response to urbanization. Similarly, we found that in the common garden, urban plants were less variable in mating system allocation than rural ones. This more uniform response to our environmental conditions is a further indication that mating system allocation in urban plants is highly plastic. Our findings of both a low outcrossing to selfing ratio in the greenhouse and a lower CV of urban than rural plants in situ suggest that urbanization has increased plasticity in mating system allocation.

### 4.3 Are Observed Differences in Flowering Phenology and Mating System Allocation due to the Timing and/or Probability Transitioning Among of Key Life History Events?

Examination of germination trait differences in urban and rural habitats is an area of research that is somewhat lacking (Piana et al. 2019). There is some evidence to suggest that differences in germination proportion between urban and rural populations tend to be relatively small, or altogether nonexistent. For example, Kostanecki et al. (2021) found that urban *Ambrosia artemisiifolia* populations had a 7% advantage in germination proportion, although this was not a statistically significant result. Yakub and Tiffin (2017) found that *Lepidium virginicum* germination proportion was high regardless of population origin (averaging 91% germination). Less evidence is available for urban effects on germination phenology; however, Galloway (2001) found that differing parental environments were strongly influential on germination time in the annual *Campanula americana* . Such differences in maternal environment have been shown to affect germination rates of *I. capensis* (Maruyama et al. 2016). Year‐to‐year variability in maternal effects is likely intensified in *I. capensis* due to lack of a persistent seed bank, in which there is no overlap between generations (Schemske 1978; Steets et al. 2007). In our study, germination proportion from both urban and rural populations was high, with miniscule (1%) recruitment differences. In contrast, we found that the timing of germination varied between population types among years, indicating that differences in contemporaneous maternal environment are as influential as urban–rural differences. While not measured directly for our populations, prevailing environmental conditions in Pennsylvania, USA (e.g., temperature and rainfall) differed between the 2 years of seed collection (i.e., 2022 was hotter and wetter than 2023; NRCC 2025). It is therefore likely that the timing of germination is influenced by heterogeneous selection across generations.

**FIGURE A2:**
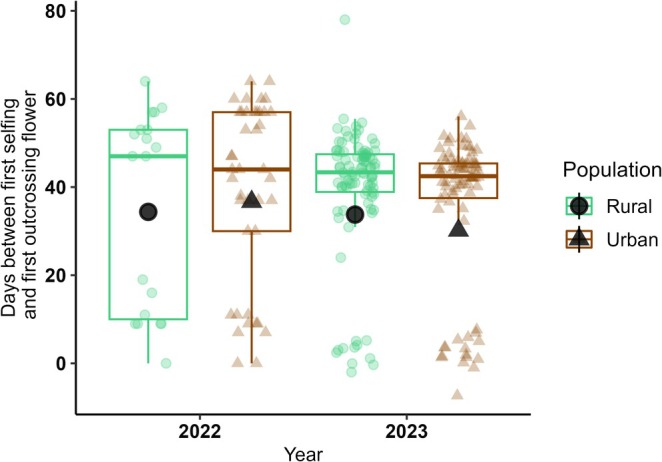
Boxplots overlayed with raw data of days between onset of selfing and onset of outcrossing flower production for rural (green circles) and urban (orange triangles) *I. capensis* in two different years of common gardens. Estimated marginal means (±SE) are shown as black symbols with error bars. Mean time between production of selfing and outcrossing flowers was more variable in 2022 than in 2023, but did not differ by year (*p* = 0.25), between population types (*p* = 0.71) or the interaction between population type and year (*p* = 0.92).

Flowering phenology has been found to be influenced by, and genetically linked to, germination phenology in several species (Chiang et al. 2009; Donohue et al. 2010; Wilczek et al. 2010; Galloway et al. 2018; Rubin and Friedman 2018). Greater sensitivity to developmental phenology can be adaptive in environments which tend to be more variable (e.g., due to climate change, Wilczek et al. 2010). While it has been well‐supported that the timing of germination directly influences downstream phenology, including flower production in other species (Donohue et al. 2002; Wilczek et al. 2010; Galloway et al. 2018; Rubin and Friedman 2018; Gremer et al. 2019; Schmitz et al. 2023), no other studies have reported a difference in sensitivity between urban and rural populations. We found that the onset of selfing covaried strongly with germination phenology. Earlier investment in selfing flowers may be further evidence for reproductive assurance in urban populations, investing in low‐cost flowers before the environment becomes too stressful as summer sets in (Heschel and Riginos 2005; de Barros Ruas et al. 2022). In contrast to selfing flower production, the onset of outcrossing phenology may be more plastic, responding to contemporaneous environmental cues as seen in other summer‐flowering species (e.g., Williams et al. 2008; Sexton et al. 2023). We found that outcrossing onset was unresponsive to changes in germination phenology. Additionally, outcrossing onset was not influenced by selfing onset (Appendix D, Table A6, Figure A2). It should be noted that although the onset of selfing and outcrossing flower production did not have the same signal of advancement as peak flowering phenology in urban plants, these results are not contradictory. Urban populations of summer‐flowering plants have been shown to produce more flowers at onset, advancing peak flowering relative to rural populations which produce fewer flowers in tandem with onset (Jochner and Menzel 2015; Davis et al. 2016; Sexton et al. 2023).

Selfing flowers are often considered a mode of reproductive assurance, and the increased likelihood of production in urban plants may reflect a history of unreliable external pollination (Eckert et al. 2010; Ushimaru et al. 2014; Rivkin and Johnson 2022; Acoca‐Pidolle et al. 2023). Additionally, greater likelihood of outcrossing flower production in urban plants has been shown to be an adaptation by urban populations to take advantage of a sparse pollinator resource (Thomann et al. 2013; Panique and Caruso 2019; Rivkin et al. 2020; Rodger et al. 2021). While records of pollinator visitation would be needed to confirm both of these predictions for *I. capensis* , we found that urban plants were more likely to produce *both* selfing and outcrossing flowers when compared to rural plants under common garden conditions (Appendix C, Figure A1B,C). Additionally, we found that both urban and rural plants were more likely to produce a selfing than an outcrossing flower. This result may derive from the fact that selfing flowers are initiated first in *I. capensis* regardless of pollinator presence (Schemske 1978; Paoletti and Holsinger 2001; Steets et al. 2007; Zhao and Schoen 2022).

Our interpretation of trait expression change in direct response to the urban environment is tempered by the fact that we did not reciprocally transplant our populations in situ, assess fitness, nor adaptation directly (Lambert et al. 2021). However, a strong signal of urban influence was maintained on floral traits, such as flowering phenology, when grown under controlled conditions. One can speculate that increased trait plasticity, earlier onset of selfing flower production with earlier germination phenology, and greater early season allocation to outcrossing may represent adaptations to both ensure reproduction and take advantage of pollinator rarity that often pervade urban environments. Future studies are needed to confirm both the genetic basis and adaptive nature of phenotypic differences between urban and rural *I. capensis* . As anthropogenic stressors continue to threaten both species interactions and biodiversity, plants with the flexibility to alter floral traits are more likely to limit fitness losses. Future work should examine the influence of interactions with other anthropogenic stressors on the phenotype and fitness of urban plants.

## Author Contributions

**Aiden M. Stanley:** conceptualization (lead), data curation (lead), formal analysis (lead), investigation (lead), methodology (lead), visualization (lead), writing – original draft (lead), writing – review and editing (equal). **Tia‐Lynn Ashman:** conceptualization (supporting), formal analysis (supporting), supervision (lead), writing – review and editing (equal).

## Conflicts of Interest

The authors declare no conflicts of interest.

## Data Availability

All data and code for this research article are available at the following DOI: https://www.doi.org/10.5281/zenodo.15040420.

## Appendix A Table of Eighteen Populations of *Impatiens capensis*

**TABLE A1:** Description of 18 Pennsylvania, USA populations of *Impatiens capensis* from this study, sorted by percent impervious surface calculated in a 5 km radius around population centroid.

Population ID	Population type	Latitude	Longitude	Percent impervious surface	Average percent canopy cover in 2021	# Seeds for 2022 cohort	# Seeds for 2023 cohort
**1**	Rural	40.20012694	−75.7900411	5.38	0.47	160	120
**2**	Rural	40.50710204	−80.3625274	7.45	NA	43	121
**3**	Rural	41.57883128	−75.7105473	7.59	NA	169	119
**4**	Rural	40.07348648	−76.8860287	8.81	0.39	158	123
5	Rural	40.93902772	−80.0927771	9.36	0.57	0	0
**6**	Rural	41.6425062	−80.4285695	9.7	0.46	121	120
7	Rural	41.8154106	−77.0826988	10.56	NA	0	0
8	Rural	41.55953783	−75.7076687	13.5	NA	0	0
9	Rural	41.06121081	−80.034941	13.56	0	0	0
10	Rural	40.24598386	−79.2461988	14.34	0.46	0	0
11	Rural	41.87277233	−80.121249	14.53	0.46	0	0
**12**	Urban	40.32677958	−76.7856686	56.97	0.06	201	122
13	Urban	41.40676131	−75.6369803	62.58	0.85	0	0
**14**	Urban	40.530391	−79.948118	65.41	0.79	40	121
**15**	Urban	41.2526353	−75.8829226	65.58	0.39	70	120
**16**	Urban	40.43612484	−79.9457865	84.9	0.44	64	121
17	Urban	40.43154139	−79.8997116	89.87	0.55	0	0
**18** ^a^	Urban	41.24575524	−80.4835382	55.4	NA	104	121

*Note:* Population type (rural or urban), GPS coordinates, percent impervious surface, and average percent canopy cover are reported for 17 field sites characterized in 2021. Populations from which seeds were collected for greenhouse studies are in bold.

^a^ Additional seed collection site not characterized in 2021.

## Appendix B Planned Contrast Tables From Significant Interaction Terms in Models for In Situ Flowering Phenology, Common Garden Germination Probability and Phenology, and Selfing Onset

**TABLE A2:** In situ flowering phenology planned contrasts with Bonferroni correction for multiple comparisons.

Contrast	Estimate	SE	df	*t*‐ratio	*p*
Rural early–Urban early	−0.793	0.298	40.6	−2.656	0.045
Rural early–Rural late	−0.718	0.224	104.2	−3.210	0.0071
Urban early–Urban late	0.385	0.496	31.6	0.776	1.0
Rural late–Urban late	0.310	0.459	28.4	0.675	1.0

**TABLE A3:** Greenhouse common garden germination probability planned contrasts of binomial model with Bonferroni correction for multiple comparisons.

Contrast	Estimate	SE	df	*z*‐ratio	*p*
Rural 2022–Urban 2022	−2.493	0.986	Inf	−2.528	0.046
Rural 2022–Rural 2023	−0.812	0.359	Inf	−2.260	0.095
Urban 2022–Urban 2023	2.777	0.6	Inf	4.629	< 0.0001
Rural 2023–Urban 2023	1.096	0.832	Inf	1.317	0.75

**TABLE A4:** Greenhouse common garden germination phenology planned contrasts with Bonferroni correction for multiple comparisons.

Contrast	Estimate	SE	df	*t*‐ratio	*p*
Rural 2022–Urban 2022	4.62	4.193	12.8	1.101	1.0
Rural 2022–Rural 2023	1.01	0.465	2252.1	2.173	0.12
Urban 2022–Urban 2023	−5.35	0.515	2253.1	−10.382	< 0.0001
Rural 2023–Urban 2023	−1.75	4.188	12.7	−0.417	1.0

**TABLE A5:** Greenhouse common garden selfing onset phenology planned contrasts with Bonferroni correction for multiple comparisons.

Contrast	Estimate	SE	df	*t*‐ratio	*p*
Rural 2022–Urban 2022	5.21	8.12	13.9	0.641	1.0
Rural 2022–Rural 2023	2.33	6.04	13.2	0.385	1.0
Urban 2022–Urban 2023	−5.74	6.03	12.9	−0.952	1.0
Rural 2023–Urban 2023	−2.86	7.96	12.8	−0.359	1.0

## Appendix D Time From Selfing Onset to Outcrossing Onset in Common Garden *I. capensis*

**TABLE A6:** Fixed effects for linear mixed models of days between selfing and outcrossing onset.

Response variable	Parameters	*X* ^2^	df	*p*
Days between selfing and outcrossing onset	Population type	0.141	1	0.7
Pseudo‐*R* ^2^ = 0.280	Year	0.009	1	0.9
*N* = 236	Population type*year	1.336	1	0.2

*Note:* Pseudo‐*R* ^2^ and sample size (*N*) are shown beneath the response variable.
